# Levetiracetam and Rhabdomyolysis: A Rare but Clinically Significant Adverse Effect

**DOI:** 10.7759/cureus.105306

**Published:** 2026-03-16

**Authors:** Pricilla A Ababio, Felix Wireko, Mekdem Bisrat, Anand Deonarine, Rawan Elkomi, Nicholas Azinge

**Affiliations:** 1 Internal Medicine, Howard University Hospital, Washington, DC, USA; 2 Internal medicine, Howard University Hospital, Washington, DC, USA

**Keywords:** antiepileptic drug toxicity, creatine phosphokinase elevation, dose-dependent adverse effect, levetiracetam, rhabdomyolysis

## Abstract

Levetiracetam (Keppra) is an antiepileptic medication known for its relatively safe side effect profile. While rhabdomyolysis represents an uncommon adverse reaction to Keppra, with only limited case reports in the literature, the relationship between levetiracetam dosage and rhabdomyolysis risk remains poorly characterized. This case report describes a patient who developed rhabdomyolysis in apparent correlation with levetiracetam dose.

We present a 29-year-old African American male with no significant past medical history who presented to the emergency department following a witnessed seizure. He had experienced two similar episodes several months prior, for which levetiracetam 500 mg twice daily was initiated. The patient reported full adherence to his prescribed medication regimen. Physical examination revealed no significant abnormalities aside from evidence of tongue laceration. Laboratory investigations demonstrated therapeutic levetiracetam levels (26 mcg/mL), creatine phosphokinase (CPK) of 2,835 IU/L, and mildly elevated serum creatinine (1.27 mg/dL). The levetiracetam dosage was subsequently increased to 750 mg twice daily.

On day 1 following dose escalation, CPK levels nearly quadrupled to 10,116 IU/L despite aggressive fluid resuscitation, and further rose to 14,400 IU/L on day 2 with concurrent worsening of renal function. Given concern that the increased levetiracetam dose may be contributory, the medication was withheld for one day and then resumed at the original home dose. Despite continued fluid resuscitation, CPK levels continued to rise, reaching a peak of 22,904 IU/L on day 5 before beginning to decline on day 6. By day 7 of hospitalization, CPK levels had decreased substantially. The patient was discharged on day 8 once CPK levels had reached a nadir and was referred to neurology for ongoing management.

## Introduction

Levetiracetam (Keppra) is a second-generation antiepileptic drug approved by the FDA in 1999 for generalized and partial seizures, with a unique mechanism of action involving binding to synaptic vesicle protein SV2A to modulate neurotransmitter release - distinguishing it from older agents that target ion channels or GABAergic transmission [1]. The drug offers several pharmacokinetic advantages, including near-complete bioavailability, minimal protein binding (<10%), and no hepatic cytochrome P450 interactions, making it well-suited for polypharmacy patients [2]. Because approximately 66% is renally eliminated unchanged, dose adjustment is required in renal impairment [2]. Typical dosing starts at 1,000 mg/day and may be titrated to 3,000 mg/day, with common side effects including somnolence, dizziness, and behavioral changes such as irritability and depression occurring in 5-13% of patients [3].

Though generally well-tolerated, levetiracetam carries rare but serious risks including Stevens-Johnson syndrome, hematologic abnormalities, and rhabdomyolysis - a potentially life-threatening syndrome involving skeletal muscle breakdown and release of intracellular components such as myoglobin and creatine phosphokinase (CPK) into circulation [4, 5]. Clinical presentation ranges from asymptomatic enzyme elevations to acute kidney injury, hyperkalemia, and cardiac arrhythmias, with CPK levels above 1,000 IU/L serving as the primary diagnostic threshold [5]. While levetiracetam is widely regarded as one of the safer antiepileptic agents, rhabdomyolysis has emerged as an uncommon yet serious adverse reaction. To date, only a limited number of cases have been documented in the literature, underscoring the rarity of this association.

## Case presentation

A 29-year-old African American male with no significant past medical history presented to the emergency department (ED) after a witnessed seizure by his significant other. He had two similar presentations a few months prior and was started on levetiracetam 500 mg twice a day. The patient claimed full compliance with his medication. Physical examination was unremarkable except for a noted tongue bite. Laboratory studies were significant for levetiracetam levels within the therapeutic range 26 mcg/mL (Normal range: 12-46 mcg/mL) and CPK of 2,835 IU/L (Normal range: Adult males: ~50-335 IU/L, Adult females: ~40-170 IU/L) with mildly elevated creatinine levels 1.27 mg/dL (Normal range: Men: 0.7-1.3 mg/dL, Women: 0.6-1.1 mg/dL). The Keppra dose was increased to 750 mg twice a day. On day 1 of Keppra administration, CPK levels had almost quadrupled despite fluid resuscitation to 10,116 IU/L and further increased to 14,400 IU/L on day 2 with worsening creatinine levels. The escalation of the levetiracetam dose was suspected as a contributing factor, and the temporal relationship between drug administration and CPK trends is illustrated in the table below. CPK levels, however, increased despite fluid resuscitation until they reached a peak of 22,904 on day 5 and started declining on day 6. Levels decreased significantly on day 7 of admission. The patient was discharged on day 8 when CPK levels came to a nadir and was referred to the neurologist for further management (Table 1).

**Table 1:** Hospital day and level of creatinine kinase

Days	Creatinine kinase (IU/L)	Creatinine (mg/dL)	Levetiracetam
0	2835	1.27	Drug dose increased, 750 mg BID
1	10116	1.73	_
2	14400	1.48	Drug was held
5	22904	1.95	_
6	12849	1.08	_
7	2350	1.12	Drug was reintroduced, 500 mg BID

## Discussion

Rhabdomyolysis results from skeletal muscle necrosis with the release of intracellular contents including myoglobin, CPK, lactate dehydrogenase, and electrolytes into the circulation [5]. Presentation ranges from asymptomatic CPK elevations, as seen in our patient, to severe acute kidney injury with myalgias, muscle weakness, and electrolyte imbalances [5]. The most serious complication is acute kidney injury, occurring in approximately 15-50% of cases through direct nephrotoxicity from myoglobin, intratubular cast formation, renal vasoconstriction, and oxidative injury [5].

While seizures are a well-recognized cause of rhabdomyolysis, an incommensurate rise in CPK with worsening renal function should prompt consideration of alternative etiologies. Brigo et al. demonstrated that CPK increases following seizures are typically mild, with mean elevations less than 1,000 IU/L and peaks occurring between 36 and 96 hours post-ictally [6]. Even in status epilepticus, CPK levels rarely exceed 10,000 IU/L and typically normalize within 3-5 days.

Our patient's CPK peak of 22,904 IU/L on day 5 substantially exceeds typical seizure-related elevations and occurred beyond the characteristic 36-96 hour window. The continued CPK rise despite the absence of recurrent seizures.

The dose-dependent nature is supported by key observations. First, the patient tolerated levetiracetam 500 mg twice daily for several months without adverse effects. The dramatic four-fold CPK increase to 10,116 IU/L within 24 hours of dose escalation to 750 mg twice daily, despite aggressive fluid resuscitation and absence of additional seizures, strongly implicates the higher dose. Second, although CPK continued rising transiently after dose reduction, peaking on day 5, this likely reflects cumulative muscle damage from higher dose exposure combined with levetiracetam's pharmacokinetic washout period. The half-life of 6-8 hours can be prolonged with renal impairment, as present in our patient.

Notably, the 750 mg twice daily dose remained within FDA guidelines despite worsening renal function, suggesting dose-dependent effects may occur even within standard therapeutic ranges, potentially reflecting an idiosyncratic reaction with dose-related severity.

The mechanism remains incompletely understood. Levetiracetam binds to synaptic vesicle protein SV2A, present in both brain tissue and skeletal muscle [1]. Interaction with skeletal muscle SV2A could disrupt vesicle trafficking and calcium homeostasis, potentially leading to cellular dysfunction and muscle fiber breakdown at higher concentrations. Alternative mechanisms may involve mitochondrial dysfunction or genetic polymorphisms in drug-metabolizing enzymes predisposing certain individuals to myotoxicity.

Previous case reports document levetiracetam-induced rhabdomyolysis at various doses. Rastogi et al. reported rhabdomyolysis with CPK of 2,400 IU/L two weeks after initiating levetiracetam 500 mg twice daily, with improvement following discontinuation [7]. However, no prior reports have documented dose-dependent relationships.

Clinicians should maintain awareness of this rare complication, particularly during dose titration. Management should include dose reduction or discontinuation when feasible, aggressive intravenous fluid resuscitation, electrolyte correction, and close renal function monitoring [8]. Limitations include inability to definitively prove causation, lack of muscle biopsy, and absence of genetic testing. Despite these limitations, the temporal relationship between dose escalation and CPK elevation and clinical course strongly support a dose-dependent drug effect.

## Conclusions

Few cases of Keppra-induced rhabdomyolysis have been reported. However, there is none reported about the dose dependence of Keppra causing rhabdomyolysis. Not much is still known about the mechanism for this rare side effect, and the mechanism for this adverse effect still remains unknown. More advanced studies are needed to evaluate the effect of Keppra on skeletal muscles and how increasing doses play a role in rhabdomyolysis.

This case highlights the importance of considering dose-dependent drug-induced rhabdomyolysis in patients on levetiracetam presenting with disproportionately elevated CPK levels. The temporal correlation between dose escalation and progressive CPK elevation, combined with features atypical for seizure-related rhabdomyolysis, underscores the need for heightened clinical awareness. Clinicians should maintain a low threshold for evaluating muscle enzyme levels in patients undergoing levetiracetam dose titration, particularly those with concurrent risk factors. Further research, including prospective studies and mechanistic investigations, is warranted to better understand this rare but potentially serious adverse effect and identify populations at increased risk.
